# Health Resorts as an integrated community healthcare model for fibromyalgia syndrome: a strategic SWOT analysis

**DOI:** 10.3389/fmed.2026.1736755

**Published:** 2026-05-14

**Authors:** Gianluca Regazzo, Francesco Piccione, Aristide Roberto Gravina, Stefano Masiero

**Affiliations:** 1 Rehabilitation Unit, Department of Neuroscience, University of Padua, Padua, Italy; 2 Department of Neuroscience, Physical Medicine and Rehabilitation School, University of Padua, Padua, Italy; 3 Neurorehabilitation Unit, Section of Brain Injury Rehabilitation, Hospital-University of Padua, Padua, Italy; 4 Rehabilitation Division, Piove di Sacco Hospital, Padua, Italy

**Keywords:** balneotherapy, fibromyalgia, Health Resorts, rehabilitation, SWOT analysis

## Abstract

**Background:**

Fibromyalgia syndrome (FMS) is a prevalent and disabling chronic pain condition associated with substantial socioeconomic burden and frequent dissatisfaction with conventional care. This supports the need for innovative community-based models. Health Resort facilities deliver multi-component, multidisciplinary programs (e.g., balneotherapy/aquatic exercise, structured exercise, education, and supportive environments) and may represent a promising setting for FMS management.

**Methods:**

This study aims to systematically evaluate the strategic potential of Health Resorts as an integrated community healthcare model for FMS using a SWOT (Strengths, Weaknesses, Opportunities, Threats) analysis. Factors were identified and synthesized through a targeted narrative search of PubMed and a structured analysis of relevant national and international policy/guidance documents. A strategic analysis was also performed to formulate actionable recommendations by combining internal and external factors.

**Results:**

Strengths included the ability to operationalize integrated biopsychosocial, patient-centered care within an immersive environment, and a potentially decentralized access model. Weaknesses included limited validation of standardized integrated protocols, heterogeneity across facilities, and suboptimal integration with public healthcare systems. Evidence for cost-effectiveness remains hypothesized pending dedicated economic evaluations.

**Conclusion:**

Health Resorts offer a promising paradigm for FMS management, shifting from fragmented treatment to an integrated, community-based approach. However, realizing this potential requires addressing critical weaknesses, particularly the need for a stronger evidence base, standardization of protocols, and improved integration with national health services.

## Introduction

Fibromyalgia syndrome (FMS) is a complex chronic pain disorder characterized by widespread musculoskeletal pain, profound fatigue, non-restorative sleep, and cognitive disturbances, leading to significant impairment in functionality and quality of life (1). Globally, FMS affects an estimated 2.7% of the population, with prevalence in Italy at approximately 1.5%–2%, corresponding to nearly 900,000 individuals (2, 3). This condition causes a severely diminished quality of life and chronic suffering, with a negative impact on patients’ physical health, psychological well-being, and social functioning (4).

From a socioeconomic standpoint, fibromyalgia causes a severe impact on healthcare systems, encompassing both high direct and indirect costs (5). Direct costs are driven by high healthcare resource utilization, including frequent physician consultations, extensive pharmacotherapy, diagnostic testing, and physical therapies. The largest economic impact, however, often arises from indirect costs, which manifest as major productivity losses (6). FMS patients frequently report low satisfaction with conventional medical care and difficulties in accessing timely, comprehensive treatment (7).

The combination of high prevalence, significant socioeconomic costs, and the limitations of current care models underscores the need for more effective and cost-efficient therapeutic strategies and settings for FMS patients. Recent literature shows that Health Resort facilities represent a promising solution for managing patients with chronic disease, as for OA patients, lymphedema and rheumatoid arthritis, among others (8–10).

By immersing patients in a health-oriented community, these facilities can provide multidisciplinary, integrated treatments for the long-term management of symptoms and the prevention of exacerbations. However, while the clinical efficacy of Health Resorts interventions in FMS, such as balneotherapy, is supported by growing evidence, a strategic evaluation of its appropriateness as a comprehensive, integrated setting for community-based FMS care is currently lacking in the scientific literature (11). While defined intervention as balneotherapy and aquatic exercise have with growing evidence for symptomatic benefit in FMS, there is a need to evaluate whether Health Resorts can function as a community-based care pathway aligned with integrated care principles, and what contextual factors might enable or hinder implementation. In this context, Health Resorts refers to an organizational and service-delivery framework in which multiple interventions are coordinated in a structured, immersive setting.

This study utilizes a SWOT (Strengths, Weaknesses, Opportunities, Threats) analysis to systematically evaluate the potential of Health Resort facilities for patients with FMS. The analysis aims to identify key internal and external factors and to propose strategic actions for the model’s development, implementation, and dissemination within modern healthcare systems.

## Materials and methods

**FIGURE 1:**
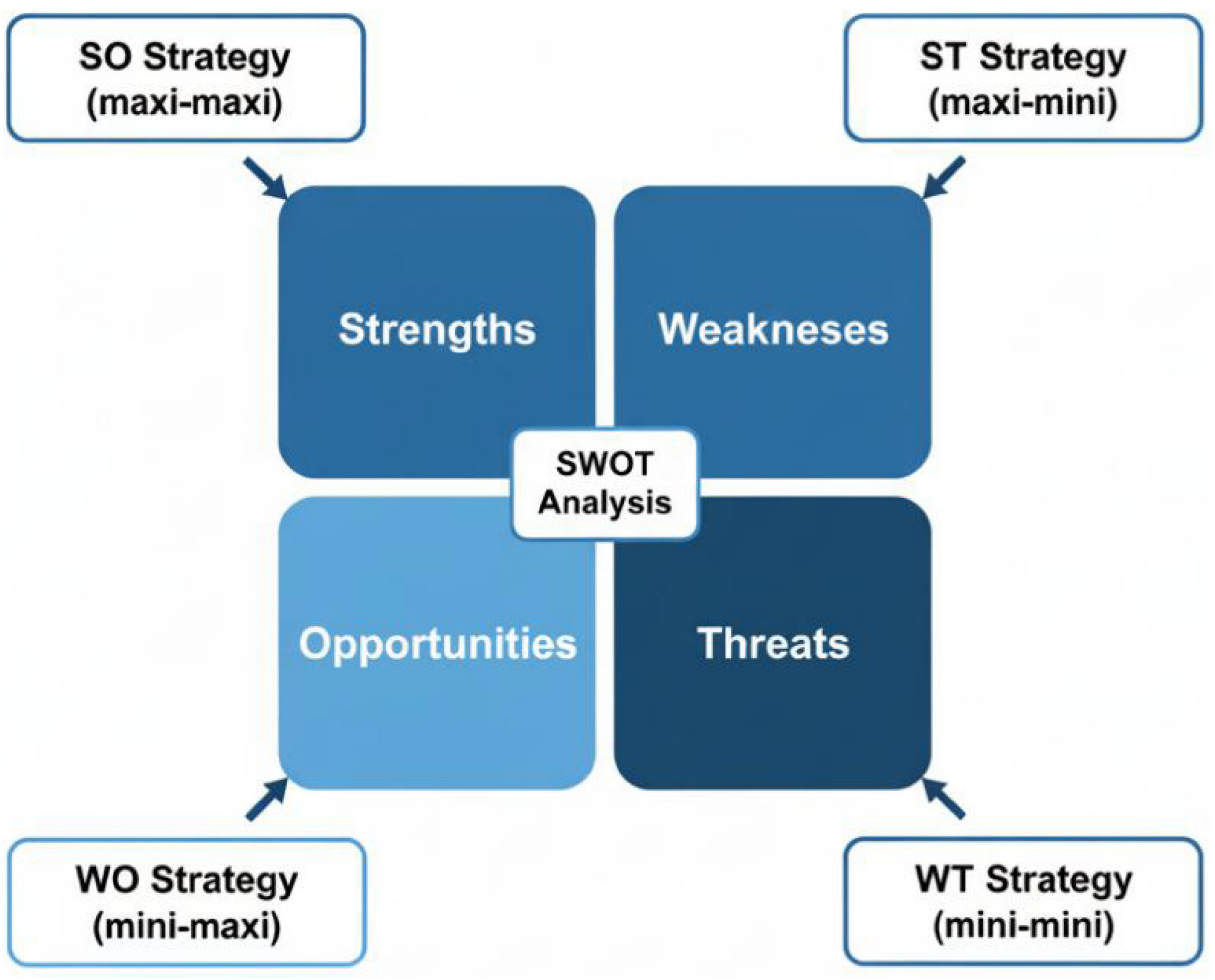
Strengths, Weaknesses, Opportunities, Threats (SWOT) analysis.

Strengths, Weaknesses, Opportunities, Threats analysis is an assessment of an organization’s internal strengths (S) and weaknesses (W), as well as its opportunities (O) for growth and improvement, and the threats (T) posed by the external environment (Figure 1). Originally developed for use in various industries, it is increasingly being applied in healthcare, where it can be a robust and valuable methodology for evaluating new treatments and proposals.

We conducted a targeted narrative literature search in PubMed (MEDLINE) to identify evidence informing the SWOT analysis of Health Resorts as an integrated community-based care setting for fibromyalgia syndrome (FMS). Search concepts were organized into four domains: (i) FMS management; (ii) water- and spa-based interventions (balneotherapy, hydrotherapy, hydrokinesitherapy, aquatic exercise/therapy, spa therapy, thermal medicine, hot springs); (iii) the organizational setting (health resort, spa/thermal resort); and (iv) care delivery models (integrated care, community-based care, decentralized care, chronic care pathways, reimbursement/financing, implementation). We prioritized human adult studies and included randomized and non-randomized clinical studies, systematic reviews/meta-analyses, clinical guidelines/consensus statements, and health-services research pertinent to access, continuity, and cost/sustainability. Reference lists of key reviews and guidelines were screened to capture additional eligible records. In parallel, we performed a structured review of national and international policy and guidance documents (e.g., WHO and national health plans) addressing integrated and community chronic care, decentralization, and reimbursement frameworks; documents were selected for institutional authority and topical relevance and analyzed via qualitative content extraction. Evidence from the scientific and policy literature was then mapped to predefined SWOT categories (Strengths, Weaknesses, Opportunities, Threats) using operational definitions (internal attributes vs. external determinants) to generate the matrix and to inform subsequent strategic matching (SO, ST, WO, WT).

## Results

The SWOT analysis identified key internal and external factors influencing the potential of Health Resorts as a setting for long-term care of FMS patients. The complete findings are detailed in Table 1.

**TABLE 1:** Strengths, Weaknesses, Opportunities, Threats (SWOT) analysis of the Health Resort model for FMS management.

Strengths (internal, positive)	Weaknesses (internal, negative)	Opportunities (external, positive)	Threats (external, negative)
**S1. Decentralized care model** Health Resorts provide decentralized healthcare services within patients’ communities, improving accessibility and reducing barriers to care (11, 27).	**W1. Limited scientific validation** Further large-scale clinical trials are required to validate standardized integrated treatment protocols (16).	**O1. High prevalence and costs of FMS** The socioeconomic burden of FMS drives the institutional need for innovative effective and cost-efficient models (5, 7).	**T1. Economic and financial threats** Declining resource allocation to public healthcare systems could impact the long-term financial sustainability of the model (23, 24).
**S2. Cost-effectiveness** Leveraging Health Resort offers a cost-effective alternative to traditional settings, potentially reducing the financial burden on public health systems (28, 29).	**W2. Heterogeneity of Health Resorts** The variability of Health Resorts in terms of medical infrastructure and specialized personnel is a key weakness (17).	**O2. Favorable normative landscape** Global and national health policies emphasize the adoption of community-based and decentralized care (21, 22).	**T2. Poor awareness among providers** Limited awareness of Health Resorts potential among the medical community and the general population (25).
**S3. Holistic and integrated care** Health Resorts facilitates the implementation of a biopsychosocial approach by integrating multiple therapeutic interventions in a single setting to address the physical, psychological, and social dimensions of FMS (12).	**W3. Individual cost** Inadequate reimbursement from national health services for Health Resort treatments creates a significant financial barrier for many FMS patients (18, 19).	**O3. Partnerships with institutions** Ongoing collaborations between Health Resorts, public entities and academic institutions aims to enhance research, secure funding, and improve accessibility (30, 31).	**T3. Resistance to change** Conventional healthcare providers and policymakers may exhibit resistance to integrating models of care delivered outside traditional hospital settings (26).
**S4. Health-promoting environment** The immersive and structured Health Resort environment promotes patient education and the adoption of self-management, which are critical for patients empowerment (13–15)	**W4. Poor integration with healthcare systems** A lack of standardized communication and shared protocols between public providers and Health Resorts compromises care continuity (20).	**O4. Integration with digital health technologies** A significant opportunity exists to integrate telemedicine and mHealth applications to extend the care continuum and home monitoring of FMS patients (32, 33).	**T4. Climate and environmental risks** Environmental changes may affect the availability and quality of thermal water, impacting their therapeutic potential (34, 35).
**S5. Utilization of natural resources** Health Resorts incorporates non-pharmacological interventions (e.g., balneotherapy, mud therapy) that have been shown to be effective in managing FMS symptoms (36, 37)	**W5. Health Resorts geographical distributions** The non-uniform geographical distribution of Health Resorts could creates access disparities, creating logistical and financial difficulties on patients from distant regions (38, 39).		**T5. Market competition**: Competition from unregulated wellness centers and providers of unvalidated therapies may undermine the Health Resorts credibility (40).

Key internal strengths primarily revolve around the model’s holistic, integrated, and patient-centered approach (S3, S4), alongside its potential for cost-effectiveness by leveraging existing infrastructures (S2) (12–15). Conversely, the main internal weaknesses identified were the limited large-scale scientific validation of integrated protocols (W1), significant heterogeneity in infrastructure and expertise across facilities (W2), and poor integration with public healthcare systems (W4) (16–20).

Externally, the analysis highlighted opportunities arising from the high prevalence and socioeconomic burden of FMS, which drives the need for new care models (O1), and a favorable policy landscape supporting decentralized care (O2) (5, 7, 21, 22). Finally, significant threats include systemic economic constraints on healthcare funding (T1), poor awareness of the model’s therapeutic potential among providers and the public (T2), and institutional resistance from conventional healthcare systems (T3) (23–26).

## Discussion

In this article, we proposed the use of SWOT analysis to provide a structured framework for evaluating the potential of Health Resorts as an integrated community care model for FMS. Our findings indicate that while the model possesses significant strengths, such as its holistic, community-based approach and its cost-effectiveness, its successful implementation requires the overcoming of weaknesses as the need for greater scientific validation and better integration with the national healthcare system.

Our analysis identifies the model’s holistic and integrated nature (S3) as a core strength. This approach directly embodies the biopsychosocial model, a framework considered essential for managing complex, multifactorial conditions such as FMS (41). Health Resorts provide a unique therapeutic environment, combining conventional treatments, health promoting interventions and complementary therapies with the beneficial effects of socialization and natural resources (42). By addressing the interplay of physical symptoms, psychological well-being, and social context, Health Resorts may support more comprehensive management of FMS (43).

Unlike traditional fragmented care, the immersive environment of Health Resorts can facilitate a stronger therapeutic alliance and promote patient empowerment and educational interventions (S4), while the proximity to patients communities could facilitate continuous care, reduce waiting times, and provide opportunities for prolonged or repeated treatment cycles in a familiar setting (S1) (44, 45).

The potential for cost-effectiveness (S2) is a critical strength, particularly given the significant economic burden caused by FMS prevalence (46). Health economic models increasingly show that decentralized care, which emphasizes self-management and lower-intensity interventions, provides better value than traditional, specialist-led models in management of chronic conditions as FMS; however, for Health Resorts formal economic evaluations are limited (47). By leveraging existing infrastructure and emphasizing non-pharmacological interventions, Health Resorts could reduce direct costs (e.g., medications, specialist visits) and indirect costs (e.g., lost productivity) associated with FMS.

A final strength of Health Resort settings is the utilization of mineral waters and acquatic exercise. Scientific literature shows that balneotherapy and hydrokinesitherapy are effective non-pharmacological interventions for managing FMS symptoms (37, 48). The thermal and chemical properties of mineral water provide significant analgesic anti-inflammatory and muscle relaxant effects, while hydrokinesitherapy facilitates gentle exercise in a buoyant environment, improving functional capacity and strength with minimal joint impact (16, 49, 50).

Despite its promise, the model faces internal weaknesses, primarily the limited scientific validation of integrated protocols (W1) and the lack of standardization across facilities (W2) (51). Other significant threats are current reimbursement policies (W3) and broader economic constraints on healthcare (T1) that could limit the financial sustainability of Health Resorts. Significantly, the lack of dedicated and stable funding mechanisms, coupled with restrictive reimbursement policies and broader healthcare budget constraints, may limit scalability and equitable access.

Finally, a systemic issue is the suboptimal integration between the public healthcare system and specialized Health Resort facilities, due to the lack of shared framework and protocols (W4).

The strategic analysis of the model suggests several strategies that could be applied to improve the validation and the diffusion of Health Resorts as an appropriate settings for management of FMS. Some key actions include the strenghtening of public-private partnerships to secure funding and create broader reimbursement policies (WO1), the promotion of sustainable management of natural resources to mitigate environmental risks (ST1) and the adoption of digital health technologies to enhance cost-effectiveness (SO1).

This study presents several important limitations that must be explicitly acknowledged. First, this is a qualitative study grounded in health services research and qualitative health policy analysis, rather than a quantitative clinical or economic evaluation. The use of SWOT analysis, while useful for strategic planning and exploratory assessment, is inherently interpretative and does not allow measurement of effect sizes, comparative effectiveness, or statistical associations. The identification and prioritization of strengths, weaknesses, opportunities, and threats are influenced by the perspectives of the researchers and by contextual assumptions, which may introduce subjectivity and potential interpretive bias. Consequently, the findings should be understood as hypothesis-generating rather than confirmatory. Second, the literature review underpinning the SWOT analysis was narrative and not systematic. Although relevant international guidelines, clinical trials, and health policy documents were considered, the review did not follow a predefined systematic protocol (e.g., PRISMA criteria), did not include structured database searches with reproducible search strings, and did not apply formal risk-of-bias assessment tools. Third, several elements of the SWOT framework are strongly conditioned by the regulatory, organizational, and reimbursement structure of the Italian healthcare system. In Italy, thermal medicine and Health Resorts operate within a specific legislative framework, characterized by regional governance of healthcare services, defined reimbursement schemes for thermal treatments, and established accreditation pathways for facilities. These regulatory conditions shape both the identified strengths (e.g., existing infrastructure and partial reimbursement) and weaknesses (e.g., limited integration with Local Health Authorities and lack of standardized shared clinical pathways). Consequently, the applicability of the model to other countries may be limited, particularly in health systems with different funding mechanisms (e.g., predominantly private insurance models), absence of recognized thermal medicine services, or differing scopes of practice for allied health professionals. In summary, this study represents an exploratory qualitative assessment situated within the Italian healthcare regulatory context and supported by a narrative review of the literature. While it provides a structured strategic framework, robust empirical research, including systematic reviews, controlled clinical studies, and formal economic evaluations, is necessary before Health Resorts can be definitively positioned as an evidence-based integrated care model for FMS.

## Conclusion

Health Resorts may represent a potentially promising paradigm for the management of FMS, as they conceptually shift care from a fragmented, predominantly biomedical approach toward a more integrated, biopsychosocial, and community-oriented model. By combining non-pharmacological therapies, supervised physical activity, psychosocial support, and environmental resources within a structured setting, they align with contemporary recommendations that emphasize multimodal and patient-centered management of complex chronic pain conditions. However, this potential must be interpreted with caution. Significant weaknesses remain, particularly the limited availability of high-quality evidence specifically evaluating fully integrated Health Resort–based care pathways for FMS. While individual components of the model (exercise therapy, balneotherapy, and educational interventions) are supported by the literature, robust randomized controlled trials assessing comprehensive, standardized resort-based programs are scarce. Moreover, the heterogeneity among Health Resorts in terms of clinical protocols, professional competencies, infrastructure, and outcome measurement represents a critical limitation that may compromise reproducibility and comparability across settings. In conclusion, Health Resorts hold theoretical and contextual promise as part of an integrated community-based strategy for FMS management. Future research should prioritize high-quality comparative studies, implementation science frameworks, and real-world programs to determine whether this model can consistently deliver clinically meaningful, sustainable, and equitable benefits.

## Data Availability

The original contributions presented in this study are included in this article/supplementary material, further inquiries can be directed to the corresponding author.
